# Photosynthetic Response of Soybean and Cotton to Different Irrigation Regimes and Planting Geometries

**DOI:** 10.3389/fpls.2022.894706

**Published:** 2022-08-08

**Authors:** Srinivasa R. Pinnamaneni, Saseendran S. Anapalli, Krishna N. Reddy

**Affiliations:** ^1^Crop Production Systems Research Unit, USDA-ARS, Stoneville, MS, United States; ^2^Oak Ridge Institute for Science and Education, Oak Ridge, TN, United States; ^3^Sustainable Water Management Research Unit, USDA-ARS, Stoneville, MS, United States

**Keywords:** photosynthesis, irrigation levels, planting geometry (PG), chlorophyll fluorescence (CF), electron transport, non-photochemical quenching, light compensation point

## Abstract

Soybean [*Glycine max* (L.) Merr.] and cotton (*Gossypium hirsutum* L.) are the major row crops in the USA, and growers are tending toward the twin-row system and irrigation to increase productivity. In a 2-year study (2018 and 2019), we examined the gas exchange and chlorophyll fluorescence parameters to better understand the regulatory and adaptive mechanisms of the photosynthetic components of cotton and soybean grown under varying levels of irrigations and planting geometries in a split-plot experiment. The main plots were three irrigation regimes: (i) all furrows irrigation (AFI), (ii) alternate or skipped furrow irrigation (SFI), and iii) no irrigation or rainfed (RF), and the subplots were two planting patterns, single-row (SR) and twin-row (TR). The light response curves at vegetative and reproductive phases revealed lower photosynthesis rates in the RF crops than in AFI and SFI. A higher decrease was noticed in RF soybean for light compensation point (LCP) and light saturation point (LSP) than that of RF cotton. The decrease in the maximum assimilation rate (Amax) was higher in soybean than cotton. A decrease of 12 and 17% in Amax was observed in RF soybean while the decrease is limited to 9 and 6% in RF cotton during the 2018 and 2019 seasons, respectively. Both stomatal conductance (gs) and transpiration (E) declined under RF. The moisture deficit stress resulted in enhanced operating quantum efficiency of PSII photochemistry (ΦPSII), which is probably due to increased photorespiration. The non-photochemical quenching (NPQ), a measure of thermal dissipation of absorbed light energy, and quantum efficiency of dissipation by down-regulation (ΦNPQ) increased significantly in both crops up to 50% under RF conditions. The photochemical quenching declined by 28% in soybean and 26% in cotton. It appears soybean preferentially uses non-photochemical energy dissipation while cotton uses elevated electron transport rate (ETR) under RF conditions for light energy utilization. No significant differences among SR and TR systems were observed for LCP, LSP, AQE, Amax, gs, E, ETR, and various chlorophyll fluorescence parameters. This study reveals preferential use of non-photochemical energy dissipation in soybean while cotton uses both photochemical and non-photochemical energy dissipation to protect PSI and PSII centers and ETR, although they fall under C3 species when exposed to moisture limited environments.

## Introduction

The United States accounts for about 35.45 million ha of soybean cultivated with a production of about 240 million Mg and a productivity of 3.4 Mg ha^−1^ (Plumblee et al., 2019). Cotton is the most important natural fiber crop worldwide, with about 34 M ha cultivated in 85 countries, and the United States accounts for 4.65 million ha with a production of 19.9 million bales (Plumblee et al., 2019). In Mississippi (MS), cotton is grown on over 0.25 M ha with an estimated production of 1.46 million bales, and soybean occupied 0.79 M ha with an estimated production of 2.8 million Mg (USDA-NASS, 2020).

In the MS Delta, many soybean and cotton growers are moving away from the traditional single-row (SR) planting geometry to twin-row (TR) geometry owing to a significant gain in productivity in most of the seasons and also to reduce machinery-associated costs as TR planters and combines can be used on multiple row crops with minor adjustments keeping the cultivation costs same for SR and TR patterns (Bruns, 2011; Bellaloui et al., 2020; Pinnamaneni et al., 2020a,b, 2021). In cotton, TR pattern adoption resulted in 35 to 106 kg ha^−1^ higher lint (Reddy et al., 2009; Stephenson et al., 2011). A range of 0–23% seed yield enhancement was reported in the TR soybean compared to the SR system (Grichar, 2007; Bruns, 2011; Pinnamaneni et al., 2020b). In the lower MS Delta, the precipitation pattern was shown to have wide fluctuations within the crop season and among the seasons resulting in drought, leading to crop yield losses (Anapalli et al., 2016).

Photosynthesis is a complex biochemical and biophysical process comprising three unique components: synthesis of photosynthetic pigments, absorption of light energy and electron transport, and the Calvin cycle of carbon fixation. It is widely believed that the photosynthetic performance appears to be a very useful indicator, owing to its strong negative correlation with different environmental stresses (Massacci et al., 2008; Zhang et al., 2011; Yao et al., 2017a; Singh and Reddy, 2018; Ye et al., 2020; Poorter et al., 2022). The capacity to utilize incident light energy by the photosynthetic system for carbon assimilation generally declines under moisture deficit conditions (Gilbert et al., 2011; Yao et al., 2017a; Poorter et al., 2022). This often leads to excessive light energy absorption than required for the efficient functioning of photosynthetic reactions, resulting in photoinhibition and cell damage (Gilbert et al., 2011; Jumrani and Bhatia, 2019). However, plants have evolved innate photoprotective mechanisms such as photorespiration and thermal emission to dissipate excess light energy to protect their machinery. Besides gas exchange assessment, often, chlorophyll fluorescence parameters are measured to understand clearly the functioning of photosynthetic components as it gives additional information on PSI and PSII reaction centers' efficiencies, energy trapping and dissipation efficiencies, and photorespiration (Massacci et al., 2008; Poorter et al., 2022). Chlorophyll fluorescence analyses reveal that the quantum efficiency of PSII (ΦPSII) is closely related to the quantum efficiency of CO_2_ assimilation when photorespiration is negligible. It was reported that soybean and cotton have distinct strategies to protect photosynthetic machinery and perform photosynthesis under moisture deficit conditions, and the reproductive stage is more critical (Zhang et al., 2011). This study further showed that soybean uses non-photochemical dissipation while cotton uses electron transport flux for light energy dissipation as their mechanism of photoprotection of photosynthetic apparatus under drought. A greenhouse study on cotton and soybean subjected to water stress revealed that the photosynthesis rate declined by 46% and 73% in cotton and soybean, respectively, while non-photochemical quenching (NPQ), an indicator of the thermal dissipation of excess excitation energy increased by 197% in soybean but only 25% in cotton under severe water stress (Inamullah and Isoda, 2005). It has been demonstrated that paraheliotropic movement in soybean (Hirata et al., 1983) and diaheliotropic movement in cotton helps to avoid photoinhibition while trying to keep leaf temperature lower under water stress (Ehleringer and Forseth, 1989). Peanut showed a greater increase than cotton in leaf temperature and NPQ and a greater decrease in chlorophyll content and ΦPSII in the water stress condition. On the other hand, the water stress lowered the transpiration rate and leaf area (LA) more in cotton than in peanut (Isoda, 2010). Furthermore, planting-row spacing impacted the photosynthetic capacity of cotton due to differences in light interception, canopy architecture, and leaf area index. A wider spacing of 76 cm in cotton recorded higher photosynthesis and boll production, while soybean did not respond to alteration in-row spacing (Slattery et al., 2017; Yao et al., 2017a). Also, differences in photosynthesis rate in soybean monocropping and intercropping with corn were reported (Yao et al., 2017b). There are no studies on the effects of SR and TR on photosynthetic and chlorophyll fluorescence parameters in the literature.

The specific objective of this research was to characterize cotton and soybean photosynthetic parameters estimated from light response curves and chlorophyll fluorescence measurements in two planting patterns (PP) i.e., SR and TR planting systems that were furrow-irrigated at the following rates (i) all furrows irrigation (AFI) (ii) skipped furrow irrigation (SFI), and (iii) rainfed (RF) at Stoneville, MS. Assessing these measurements will enhance our understanding on the regulation of photosynthesis under moisture deficit environments.

## Materials and Methods

Two-year (2018–2019) field experiments were conducted at the USDA-ARS, Crop Production Systems Research Unit's research farm, in Stoneville, MS, USA (33° 42′ N, 90° 55′ W, elevation: 32 m above mean sea level). The experimental area soil type was a Dundee silt loam (fine silty, mixed, active, thermic Typic Endoaqualfs) with 0.92% organic matter, 0.46% carbon, 0.07% nitrogen, and 1.26 g cm^−3^ bulk density averaged across 60 cm soil depth. As measured in this study, the field saturated hydraulic conductivity (Kfs) of the soil ranged from 0.39 to 1.26 cm hr^−1^. Field preparation consists of deep tillage in the fall, disking in winter, and harrowing in early spring to make ridges and furrows at 102 cm spacing as described earlier (Pinnamaneni et al., 2020a). A 7300-vacuum planter (John Deere, East Moline, IL) was used to plant in the SR planting geometry while a Monosem NG+3 TR vacuum planter (A.T.I., Inc. Monosem, Lenexa, KS) was used to plant the TR planting geometry in cotton. Both planters were set to achieve a similar population density of approximately 120,000 plants ha^−1.^ Soybean SR plantings were made using an Almaco cone plot planter (Allen Machine Company, Nevada, IA), and TR plots were planted with a four-unit Monosem NG-3 (Monosem, Edwardsville, KS) twin-row vacuum planter. Both planters were set to achieve a similar overall plant population density of approximately 336,000 plants ha^−1^. Plots were maintained weed-free using both pre-emergence and postemergence herbicide programs (Pinnamaneni et al., 2020b).

The cotton cultivar “FiberMax1944GLB2,” a medium-maturing Bt-transgenic (Cry1Ac and Cry2Ab genes) variety with broad adaptation, possessing in-plant tolerance to glyphosate, and glufosinate reaches 50% flowering by 75 days, and soybean maturity group IV cultivar “31RY45 Dyna-Gro” with roundup ready trait reaching 50% flowering by 43 days were planted in two separate studies. Both cultivars are popular in MS Delta. The former is known for its fiber yield while the latter is tolerant to drought. The treatments were arranged as a split-plot design with six replications. The main plots were three irrigation regimes (a) AFI, (b) SFI, and (c) RF. Subplots consisted of two planting geometries: (a) SR, single rows evenly spaced at 102 cm centered seedbeds, and (b) TR, two rows spaced 25 cm apart on 102 cm centered seedbeds. Cotton seeds were planted on 8 May 2018 and 16 May 2019. Soybean was planted on 8 May 2018 and 2 May 2019. Each plot consisted of four SR or eight TR rows and was 40 m long. Sensors for measuring soil–matrix water potential (Irrometer Company, Inc, Riverside, CA, USA) were installed in the selected representative plots, and irrigations were scheduled based on a soil matrix potential of about −90 kPa at 45 cm soil depths, as Plumblee et al. (2019) recommended. Soybean was grown without any fertilizer while cotton received nitrogen in the form of 32% urea–ammonium nitrate, which was injected 7.5 cm deep in a split application of 112 kg N ha^−1^ 50% at planting and 50% at 35 days after planting. Average crop evapotranspiration of soybean and cotton was 546 and 552 mm, respectively, in the lower MS Delta, and annual average effective rainwater deficits for soybean and cotton were estimated to be 340 mm and 395 mm, respectively (Tang et al., 2018). In 2018, a total of 220 mm of irrigation was applied to the AFI treatments in four irrigation events while 152 mm was applied in three irrigations in 2019 for soybean, and cotton received 175 mm in five irrigation events and 152 mm in three irrigation events in 2018 and 2019, respectively. The SFI plots received 50% of AFI treatment (Pinnamaneni et al., 2020b, 2021). Irrigation was stopped at the R6 stage of growth of pod development in both years for soybean, while for cotton it was at the boll cracking stage (C5). Weather data was collected from the Stoneville AWS station (latitude:33.43122, longitude: −90.91077), Delta Research and Extension Center, Stoneville, MS. The grain yield in soybean and lint yield in cotton were estimated as reported earlier (Pinnamaneni et al., 2020a,b).

Data on light response curves and gas exchange rates were measured at vegetative (V6 in both the crops) and reproductive phases (R5 in soybean and C5, 5^th^ boll cracking in cotton) using a portable infrared (IR) gas analyzer (LI-6800, LI-COR, Lincoln, NE) equipped with a standard 2 × 3 cm leaf chamber. The dates of measurement in soybean were between 21 June and 16 July 2018 and between 11 June and 17 July 2019, while the data on cotton were collected on 30 June and 3 August 2018 and 3 July 3 and 12 August 2019 between 9 am and 3 pm. The leaf temperature was between 28 and 31 °C, while the RH was maintained at 60% and the CO_2_ concentration in the chamber was 400 mg L^−1^. Ten photosynthetic photon flux density (PPFD) levels (−2,000, 1,500, 1,000, 500, 250, 120, 60, 30,15, and 0 μmol m^−2^s^−1^) were used. Fully expanded top leaves on 3 randomly selected plants per plot were used for measurements. The leaves were allowed to adapt to each light level for 10 min before measurement. Then, after linear fitting, light compensation point (LCP), light saturation point (LSP), light-saturated net photosynthetic rate (Amax), apparent quantum efficiency (AQE), and dark respiration rate (Rd) were estimated by the method of Ye (2007).

Pre-dawn measurements of minimal fluorescence (F_o_) and maximal fluorescence (F_m_) were measured on dark adapted fully expanded top leaves according to Baker (2008). The maximum fluorescence value in the light (F${}_{\text{m}}^{\prime }$) was estimated after applying a 0.8-s saturation flash (10,000 μmol m^−2^ s^−1^). The minimal fluorescence in the light-adapted leaves (F${}_{\text{o}}^{\prime }$) was calculated according to Oxborough and Baker (Oxborough and Baker, 1997). Electron transport rate (ETR) was calculated by assuming a leaf absorption of 0.85 and a PSII:PSI ratio of 1:1.

The chlorophyll fluorescence parameters were calculated according to Rosenqvist and van Kooten (Genty et al., 1989; Rosenqvist and van Kooten, 2003; Kramer et al., 2004).


$$\begin{array}{l} Fo^{{}^{\prime }}=Fo/\left(\frac{\text{Fv}}{Fm}+\frac{\text{Fo}}{Fm^{{}^{\prime }}}\right) \end{array}$$

Operating quantum efficiency of PSII photochemistry,


$$\begin{array}{l} \Phi \text{PSII}=\frac{\text{F}{\text{m}}^{{}^{\text{′}}}-{\text{F}}^{{}^{\text{′}}}}{\text{F}{\text{m}}^{{}^{\text{′}}}} \end{array}$$

$$\begin{array}{l} \text{Photochemical quenching }\left(\text{QP}\right)\text{ as QP}=\frac{\text{F}{\text{m}}^{{}^{\text{′}}}-{\text{F}}^{{}^{\text{′}}}}{\text{F}{\text{m}}^{{}^{\text{′}}}-\text{F}{\text{o}}^{{}^{\text{′}}}} \end{array}$$

$$\begin{array}{l} \text{Non}-\text{photochemical quenching }\left(\text{NPQ}\right)\text{ was expressed as NPQ}\\ =\frac{\text{Fm}}{\text{F}{\text{m}}^{{}^{\text{′}}}}-1 \end{array}$$

$$\begin{array}{l} \text{Quantum efficiency of dissipation by down}-\text{regulation }\\ as\;\Phi NPQ=\left(\frac{F^{{}^{\prime }}}{Fm^{{}^{\prime }}}\right)-1/\left(\frac{Fm}{Fm^{{}^{\prime }}}+\;\frac{Fq^{{}^{\prime }*}Fo^{{}^{\prime }*}Fv}{Fv^{{}^{\prime }*}F^{{}^{\prime }*}Fo}\right) \end{array}$$

An analysis of variance (ANOVA) was conducted using PROC MIXED (JMP Pro v. 14.1.0 software, SAS Institute, Cary, NC). Year, PP, irrigation levels, and interactions were considered fixed effects. Replicates within a year were considered random effects. Mean comparisons were conducted by Fishers Protected LSD test, and the level of significance of P ≤ 0.05 was used. The graphs were made using Sigmaplot (Version 14.0, Systat software).

## Results and Discussion

### Seasonal Weather

**Figure 1:**
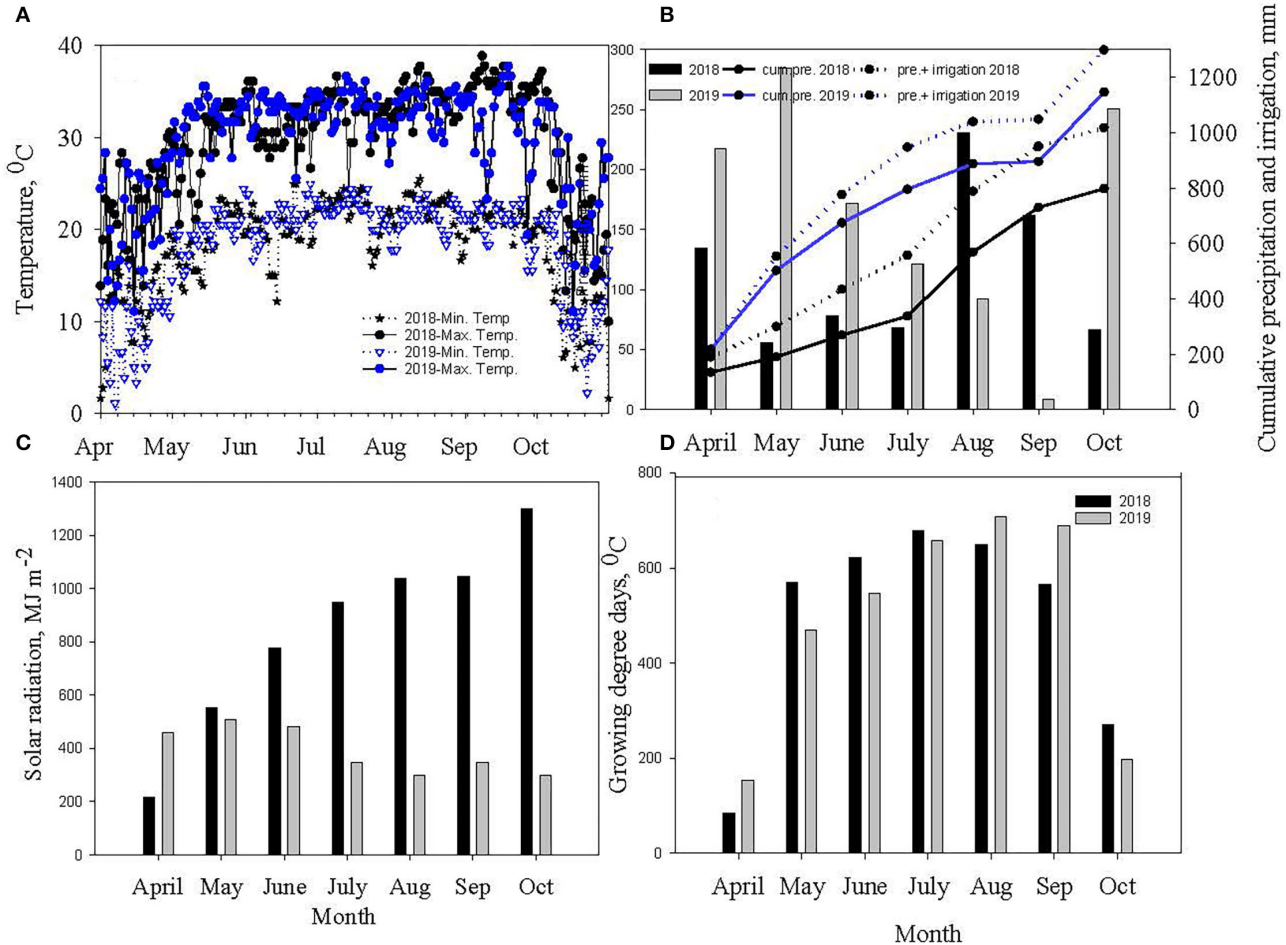
Measured **(A)** daily air temperature and **(B)** monthly precipitation, and cumulative precipitation and irrigation **(C)** monthly solar radiation, and **(D)** monthly growing degree days (GDD) for 2018 and 2019 soybean and cotton growing seasons at Stoneville, MS.

The two cropping seasons in 2018 and 2019 were vividly different in weather conditions (Figure 1). Warmer weather prevailed during reproductive growth and boll filling (July–September) in 2019 (92 GDD more than in 2018). The 2019 crop season was dry (348 mm less rainfall) and had more monthly total solar radiation of 500 MJ m^−2^ than in 2018. However, vegetative growth (May–July) in 2018 coincided with periods of lower rainfall (375 mm <2019) and higher mean minimum and maximum temperatures. Due to the impacts of contrasting weather across the two growing seasons, the analysis of variance (ANOVA) revealed that year has significant interaction with different photosynthetic parameters (Table 1).

**Table 1:** Analysis of variance (*F* and *P* values^a^) for the effect of year (Y), planting pattern (PP), and irrigation levels (I) and their interaction on soybean photosynthesis chlorophyll fluorescence parameters.

**Effect**	**DF**	**LCP**		**LSP**		**AQE**		**A**		**gs**		**E**		**ETR**		**ΦPSII**		**QP**		**NPQ**		**ΦNPQ**		**Rd**	
		**F**	**P**	**F**	**P**	**F**	**P**	**F**	**P**	**F**	**P**	**F**	**P**	**F**	**P**	**F**	**P**	**F**	**P**	**F**	**P**	**F**	**P**	**F**	**P**
Year (Y)	1	8.65	ns	44.63	ns	2.31	^*^	18.44	^*^	9.12	^*^	5.08	^**^	43.82	^**^	2.41	^*^	1.2	^*^	1.25	^*^	1.03	ns	5.62	ns
PP	1	4.68	ns	15.67	ns	1.28	ns	2.21	ns	1.32	ns	1.66	ns	10.55	ns	1.43	^*^	9.82	ns	4.11	ns	0.82	ns	1.56	ns
Y*PP	1	3.58	ns	11.52	ns	2.52	ns	1.73	ns	1.49	ns	0.56	ns	1.22	ns	0.45	ns	1.09	ns	1.09	ns	1.09	ns	1.14	ns
Irrigation (I)	2	18.74	^*^	86.94	^*^	11.24	^*^	1.77	^**^	12.76	^**^	2.56	^**^	185	ns	7.93	^**^	0.25	^**^	0.47	^**^	1.02	^**^	10.25	^**^
Y*I	2	9.65	ns	18.57	^*^	4.68	ns	21.81	^**^	7.59	^**^	1.36	^**^	3.59	^*^	8.02	^**^	0.98	ns	0.98	ns	0.98	ns	1.42	ns
PP*I	2	2.82	ns	15.69	ns	2.44	ns	0.43	ns	0.15	ns	0.66	ns	0.71	ns	0.19	ns	0.32	ns	0.24	ns	0.61	ns	1.24	ns
PP*I*Y	2	1.44	ns	6.57	ns	2.05	ns	1.22	ns	1.01	ns	0.18	ns	0.16	ns	0.59	ns	0.11	ns	0.18	ns	0.19	ns	0.47	ns
Residuals		0.35	ns	3.68	ns	0.189	ns	501		1.662		0.252		11.14		0.033		1.992		0.032		0.014		0.029	

a* *Significance at P ≤ 0.05*;

** *Significance at P ≤ 0.001; ns, not significant*.

*LCP, light compensation point (μmol m^−2^ s^−1^); LSP, light saturation point (μmol m^−2^ s^−1^); AQE, apparent quantum efficiency; Amax, Light saturated net photosynthesis (μmol CO_2_ m^−2^ s^−1^); gs, stomatal conductance (mmol H_2_O m^−2^ s^−1^); E, transpiration (mmol H_2_O m^−2^ s^−1^); ETR, electron transport rate (μmol m^−2^ s^−1^); ΦPSII, quantum efficiency of primary PSII photochemistry; QP, photochemical quenching; NPQ, non-photochemical quenching; ΦNPQ, quantum efficiency of non-photochemical quenching; R_d_, dark respiration (μmol m^−2^ s^−1^)*.

*The experiment was conducted in Stoneville, MS, USA, in 2018 and 2019*.

### Light Response Curves in Soybean

**Figure 2:**
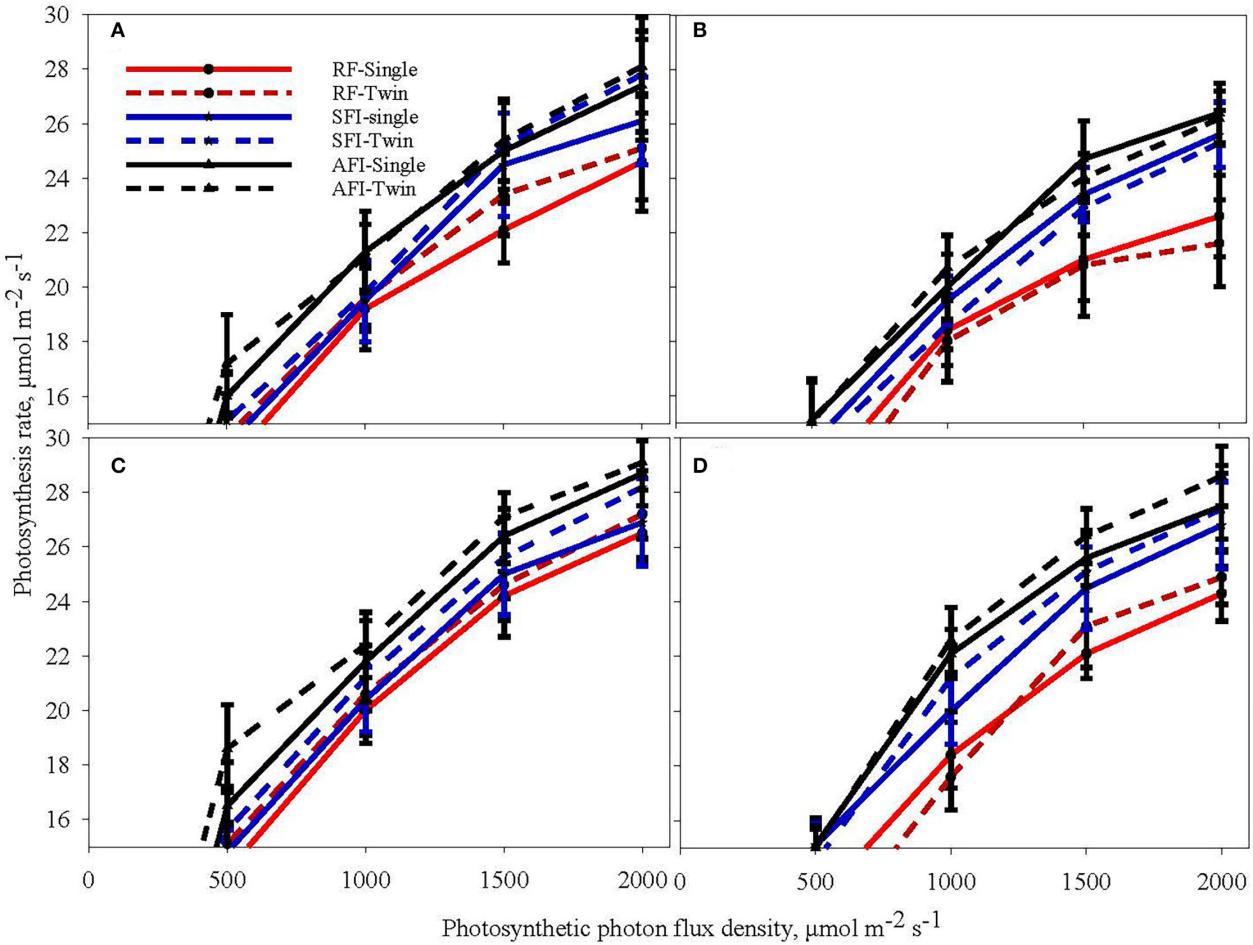
Light response curves of soybean at the sixth leaf (V6) stage **(A,B)** and beginning seed set (R5) stage **(C,D)** during the crop growing seasons in 2018 **(A,C)** and 2019 **(B,D)** at different levels of irrigation (AFI- all furrow irrigation SFI- skipped furrow irrigation and RF-rainfed) and planting geometries (SR-single row and TR-twin row).

Figure 2 shows light response curves (assimilation rate vs. the photosynthetic photon quanta flux density, PPFD) in soybean at the vegetative stage (V6) and reproductive stage (R5) for all the irrigation and planting geometry combinations for the 2018 and 2019 seasons. The CO_2_ assimilation rate is relatively lower at the reproductive stage in both the years than in the vegetative stage. Further, the figure reveals that the assimilation rates were higher in 2018 than in 2019, although comparatively less. The assimilation rates under different treatments initially increased with a rise in PPFD ranging from 0 to 2,000 μmol m^−2^s^−1^. The increasing trend subsequently plateaued and eventually reached a saturation point at 1,500 μmol m^−2^s^−1^. As the PPFD continued to increase, the light response curves of the net assimilation rate under AFI and SFI became higher than those of RF treatments. There appears to be no significant difference caused by PP on photosynthesis in both years. The TR planting system recorded a 13% higher grain yield on an average than the SR system primarily due to a 9% higher plant stand per unit area (Pinnamaneni et al., 2020b). These results suggest that both AFI and SFI positively impacted the CO_2_ assimilation rate. The RF soybean in both seasons recorded a significantly lower photosynthesis rate under saturated light conditions. These results conform to the earlier reports of reduced photosynthesis under water-stressed conditions (Zhang et al., 2011; Yao et al., 2017a; Du et al., 2020).

### Light Response Curves in Cotton

**Figure 3:**
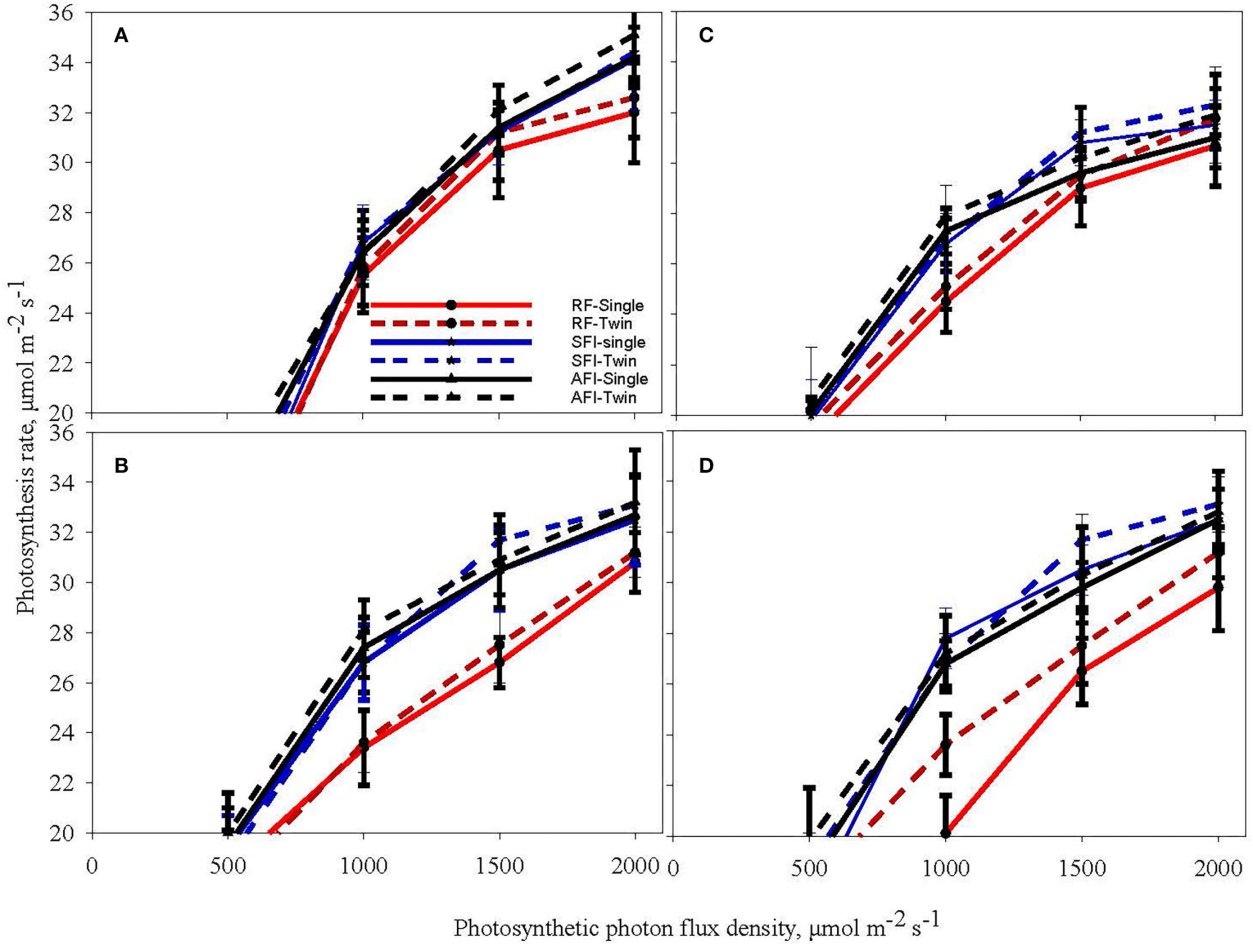
Light response curves of cotton at the sixth leaf (V6) stage **(A,B)** and boll cracking stage-C5 **(C,D)** during the crop growing seasons in 2018 **(A,C)** and 2019 **(B,D)** at different levels of irrigation (AFI- all furrow irrigation SFI- skipped furrow irrigation and RF-rainfed) and planting geometries (SR-single row and TR-twin row).

Figure 3 shows light response curves (assimilation rate vs. the photosynthetic photon flux density, PPFD) for cotton in the vegetative stage (V6) and reproductive stage (C5) for all the irrigation and planting geometry combinations for both seasons. Cotton plants exhibited a significantly higher CO_2_ assimilation rate under irrigated conditions (AFI and SFI) than in a rainfed situation in both the vegetative and reproductive stages (Figure 3). No differences were observed among SR and TR plantings for CO_2_ assimilation rate. The TR pattern recorded a 23% increased plant population per unit area over the SR system, which probably has contributed to a 15% lint yield advantage in spite of similar assimilate rates (Pinnamaneni et al., 2020a). Like soybean, the CO_2_ assimilation rate plateaued under light saturation conditions at 1500 μmol m^−2^s^−1^in cotton. The results of this study echo the observations of Massacci et al. (Massacci et al., 2008) while contradicting the findings of Yao et al. (2017a). In a study from China, Yao et al. (2017a) demonstrated that photosynthesis rate is influenced by plant spacing in the following order: wide row spacing > medium row spacing > narrow row spacing. This is probably due to differences in canopy characteristics as cotton was demonstrated to change canopy architecture *vis a vis* row spacing, cultivar differences, and plant stand establishment. It has been demonstrated that narrow-row and twin-row cotton have a higher leaf area index, which contributes to higher CO_2_ assimilation (Heitholt et al., 1992; Pettigrew, 2015; Pinnamaneni et al., 2020a).

### Photosynthesis and Chlorophyll Fluorescence Parameters at the Reproductive Stage

#### Soybean

The ANOVA revealed significant differences in photosynthetic and chlorophyll fluorescence traits such as A, gs, E, LSP, LCP, AQE, ETR, ΦPSII, NPQ, QP, ΦNPQ, and R_d_ among the treatments (Table 1). The irrigation level had significant effects on A, gs, E, LSP, LCP, ETR, ΦPSII, NPQ, QP, ΦNPQ, and R_d_, while year affected all the traits except Ø_NPQ_ and R_d_ probably due to differences in weather parameters, such as solar radiation (Figure 1C), temperature (Figure 1A), and humidity (data not shown), in spite of collecting data on non-cloudy days. None of the interactions were significant except for irrigation X year. The mean values for all the traits year-wise are presented in Table 2 as ANOVA revealed significant differences for most of the traits among the two seasons. The LCP ranged from 58 to 95 μmol m^−2^ s^−1^ in 2018 while it was between 43 and 77 μmol m^−2^ s^−1^, and RF crops recorded 42 and 59% decreases in 2018 and 2019, respectively. Similar results were earlier reported on water-stressed soybean by Zhang et al. (2011). The LSP decreased significantly in both years by 37%. The AQE in RF crops increased by 10 and 7% in 2018 and 2019, respectively. The Amax ranged between 28.84 and 29.15 μmol CO_2_ m^−2^ s^−1^ in the irrigated crop, while the range for the rainfed crop is between 25.71 and 26.01 μmol CO_2_ m^−2^ s^−1^ in 2018. It ranged between 19.77 and 22.94 μmol CO_2_ m^−2^ s^−1^ in the irrigated crop of the 2019 season, while it was between 18.31 and 19.00 μmol CO_2_ m^−2^ s^−1^ for the rainfed crop. On average, irrigated crops recorded about 12 and 17% higher photosynthesis in 2018 and 2019, respectively, compared to the rainfed crop. A similar decrease in photosynthesis under water-deficit conditions was reported earlier (Gilbert et al., 2011; Zhang et al., 2011; Cotrim et al., 2021). It is believed that decrease in LSP, LCP, and Amax, and increased AQE in soybeans under shading stress an acclimation strategy (Hussain et al., 2020). The stomatal conductance varied between 0.44 and 0.55 mmol H_2_O m^−2^ s^−1^ in 2018, while the range was from 0.45 to 0.62 mmol H_2_O m^−2^ s^−1^ in 2019. The PP did not affect this parameter in the three irrigation regimes. The gs reduced by 17% in 2018 and 24% in 2019, under RF, as stomata closure under moisture deficit conditions is widely believed to be a major physiological response to resist leaf wilting, desiccation, and plant death (Gilbert et al., 2011; Cao et al., 2017). The E levels also negatively impacted 32% and 19% in 2018 and 2019, respectively. However, the ETR was significantly higher in the rainfed crop by 6 and 9% in 2018 and 2019, respectively. These findings echo the earlier observations (Jumrani and Bhatia, 2019). Significant differences were observed in most of the chlorophyll fluorescence parameters studied among the irrigation treatments. The water deficit in RF soybean resulted in a sharp increase in ΦPSII. The rise in ΦPSII (17% in 2018 and 8% in 2019) could be attributed to the fact that moisture deficit activates the mitochondrial alternative oxidase leading to less heat dissipation, which probably delays the ΦPSII decrease (Bartoli et al., 2005). The data among AFI, SFI, and RF treatments reveals that an increase of NPQ by 34% in 2018 and 44% in 2019 under water-deficit conditions is probably a consequence of a concomitant decrease in the excitation energy trapping efficiency of PSII and the non-photochemical energy dissipation. Similarly, a significant increase of ΦNPQ in RF (30% in 2018 and 29% in 2019) was observed, revealing its role in protecting the photosynthetic apparatus from heat damage. These observations support the earlier findings where water-limited environments triggered a significant increase in NPQ and ΦNPQ than that of well-irrigated crops (Zhang et al., 2011, 2016; Cao et al., 2017). In contrast, QP levels, an indicator of the fraction of open PS II reaction centers, declined to the extent of 21% and 35% in the 2018 and 2019 seasons, respectively, in the RF crop, which is believed to affect the balance between the excitation rate and the ETR. This change may have led to a reduced state of the PSII reaction centers as ETR levels increased marginally in the RF crop (6% in 2018 and 9% in 2019). These findings confirm the previously published reports (Gilbert et al., 2011; Zhang et al., 2011, 2016; Yao et al., 2017a). The Rd is another key parameter estimating CO_2_ loss and mitochondrial respiration increased by 34 and 22% in 2018 and 2019, respectively, indicating its role in sustaining the photosynthesis rates under low gs. These results corroborates with the observations of Yao (Slattery et al., 2017; Yao et al., 2017a). Photosynthesis is a good indicator of plant growth and metabolism owing to its significant association with biomass production and grain yield (Gilbert et al., 2011). The current results of photosynthesis and chlorophyll fluorescence parameters corroborate well with previously published results, and the grain yield in different irrigation- planting geometry combinations were 4.8 Mg ha^−1^ in AFI under TR, 4.7 in Mg ha^−1^ in SFI under TR, 4.2 Mg ha^−1^ in AFI under SR, 4.1 Mg ha^−1^ each in RF under TR and SFI under SR, and 3.6 Mg ha^−1^ in RF under SR (Pinnamaneni et al., 2020b).

**Table 2:** Effect of irrigation and planting geometry on soybean and cotton photosynthetic parameters in 2018 and 2019.

**2018**	**Soybean**							**Cotton**					
	**AFI**		**SFI**		**RF**			**AFI**		**SFI**		**RF**		
	**SR**	**TR**	**SR**	**TR**	**SR**	**TR**	**LSD**	**SR**	**TR**	**SR**	**TR**	**SR**	**TR**	**LSD**
LCP	95a	82b	88b	78b	63c	58c	11	122a	108b	125a	112b	99c	95c	13
LSP	1879a	1842a	1865a	1784	1270c	1238c	65	2245a	2146b	2257a	2180b	1738c	1643d	62
AQE	0.057c	0.052e	0.055d	0.053e	0.061a	0.059b	0.004	0.059a	0.057b	0.057b	0.053c	0.051d	0.048e	0.004
Amax	29.15a	28.94a	28.84a	28.92a	25.71b	26.01b	1.01	33.22a	32.77a	33.26a	32.5a	29.12b	30.54b	1.45
R_d_	−2.98b	−2.83b	−3.42a	−3.22a	−2.4oc	−2.25c	0.24	−3.44a	−3.25a	−3.46a	−3.22ab	−3.33a	−2.48c	0.22
gs	0.51a	0.55a	0.52a	0.5a	0.45b	0.44b	0.06	0.67a	0.68a	0.64a	0.59b	0.61a	0.55b	0.06
E	2.95a	2.86a	2.87a	2.75a	2.12b	2.2b	0.17	3.91a	3.85a	3.77a	3.45b	3.35b	2.84c	0.17
ETR	224.8b	201.4b	228.1a	234.3a	238.6a	235.4a	13.22	288.6b	291.5b	284.7b	294.3b	322.1a	324.7a	19.9
ΦPSII	0.48bc	0.46c	0.5b	0.49b	0.58a	0.59a	0.04	0.56b	0.58b	0.56b	0.57b	0.65a	0.68a	0.06
qp	0.65a	0.68a	0.65a	0.66a	0.54b	0.55b	0.04	0.78a	0.77a	0.75a	0.73a	0.62b	0.59b	0.12
NPQ	0.87b	0.85b	0.9b	0.92b	1.34a	1.34a	0.1	2.1c	1.98c	2.22c	2.34b	3.19a	3.45a	0.31
ΦNPQ	0.23b	0.20b	0.19b	0.21b	0.31a	0.30a	0.07	0.15b	0.16b	0.16b	0.18b	0.29a	0.27a	0.04
**2019**	**SR**	**TR**	**SR**	**TR**	**SR**	**TR**	**LSD**	**SR**	**TR**	**SR**	**TR**	**SR**	**TR**	**LSD**
LCP	77	67	72	64	45	43	5	125	112	136	118	106	98	11
LSP	1789a	1721b	1634c	1687bc	1338d	1352d	52	2324b	2411a	2287b	2316b	1946c	1958c	54
AQE	0.059a	0.057a	0.053a	0.051b	0.06a	0.058a	0.008	0.054a	0.051a	0.053a	0.055a	0.049a	0.05a	0.009
Amax	21.67	22.94	19.77	22.71	19	18.31	1.3	34.32a	35.01a	34.15a	34.49a	32.13b	32.55b	1.85
R_d_	−3.11a	−3.34a	−2.72b	−3.26a	−2.45b	−2.65b	0.29	−3.48a	−3.4a	−3.36a	−3.34a	−2.74b	−2.85b	0.28
gs	0.56b	0.59a	0.49c	0.62a	0.46c	0.45c	0.03	0.74a	0.76a	0.71a	0.64b	0.6b	0.58b	0.05
E	3.78a	3.75a	3.04b	3.85a	3.01b	3.06b	0.13	4.25a	4.32a	4.35a	4.45a	3.54b	3.58b	0.34
ETR	196.8bc	186.9c	218.6a	202.3ab	214.6a	225.6a	19.21	254.6bc	260.5b	280.5b	276.5b	302.1a	297.6a	24.5
ΦPSII	0.43a	0.42a	0.43a	0.44a	0.49a	0.48a	0.09	0.52b	0.53b	0.55ab	0.52b	0.61a	0.63a	0.1
QP	0.74a	0.76a	0.69b	0.72a	0.56c	0.52c	0.07	0.82a	0.81a	0.83a	0.80a	0.65b	0.62b	0.11
NPQ	0.74b	0.77b	0.81b	0.72b	1.28a	1.42a	0.13	2.40b	2.26b	2.34b	2.48b	2.97a	3.07a	0.24
ΦNPQ	0.22b	0.25b	0.21b	0.24b	0.33a	0.32a	0.08	0.12b	0.12b	0.13b	0.14b	0.24b	0.27b	0.05

*LSD, Least Significant Difference test; significant at P ≤ 0.05. Means followed by the same letter within each row and crop are not significantly different. AFI, All furrow irrigation; SFI, Skip furrow irrigation; RF, rainfed; LCP, light compensation point (μmol m^−2^ s^−1^); LSP, light saturation point (μmol m^−2^ s^−1^); AQE, apparent quantum efficiency; Amax, maximum assimilation (μmol CO_2_ m^−2^ s^−1^); gs, stomatal conductance (mmol H_2_O m^−2^ s^−1^); E, transpiration (mmol H_2_O m^−2^ s^−1^); ETR, electron transport rate (μmol m^−2^ s^−1^); ΦPSII, quantum efficiency of primary PSII photochemistry; QP, photochemical quenching; NPQ, non-photochemical quenching; ΦNPQ, quantum efficiency of non-photochemical quenching; R_d_, dark respiration (μmol m^−2^ s^−1^)*.

*The experiment was conducted in Stoneville, MS*.

#### Cotton

The ANOVA revealed significant differences in photosynthetic and chlorophyll fluorescence traits such as A, gs, E, LSP, LCP, AQE, ETR, NPQ, QP, ΦNPQ, ΦPSII, and R_d_ among the treatments (Table 3). The irrigation level had significant effects on A, gs, E, LSP, LCP, AQE, ETR, NPQ, QP, ΦNPQ, and R_d_, while year affected all the traits except QP, NPQ, ΦNPQ, and R_d_. None of the interactions were significant except for irrigation X year. The mean values for all the traits year-wise are presented in Table 2 as ANOVA revealed significant differences for most of the traits among the two seasons. The LCP ranged from 95 to 125 μmol m^−2^ s^−1^ in 2018 and between 98 and 136 μmol m^−2^ s^−1^ in 2019. The RF crop recorded a 20% decrease in both seasons for LCP. A decrease of 38% in water-stressed soybean (Zhang et al., 2011) and between 41% and 72% decrease in sorghum under drought (Zhang et al., 2019) was earlier reported. However, the decrease in RF crop for LSP was 31% and 25% in 2018 and 2019, respectively. The AQE ranged from 0.048 to 0.059 in 2018 while it varied between 0.049 and 0.055 in 2019. The RF crop recorded a 14% and 11% decrease compared to irrigated crops for AQE in the 2018 and 2019 seasons, respectively. Similar levels of decrease in AQE in drought-stressed cotton were observed in a drip-irrigated study in China (Zhang et al., 2011). The Amax ranged between 32.77 and 33.26 μmol CO_2_ m^−2^ s^−1^ in the irrigated crop while the range for the RF crop is between 29.12 and 30.54 μmol CO_2_ m^−2^ s^−1^ in 2018. The Amax ranged between 34.15 and 35.01 μmol CO_2_ m^−2^ s^−1^ in the irrigated crop of the 2019 season, while the RF crop ranged between 32.13 and 32.55 μmol CO_2_ m^−2^ s^−1^. On average, irrigated crops recorded about 9% and 6% higher photosynthesis in 2018 and 2019, respectively, compared to the RF crop. A similar decrease in photosynthesis under RF conditions was reported earlier (Heitholt et al., 1992; Massacci et al., 2008; Zhang et al., 2011). The stomatal conductance varied between 0.55 and 0.68 mmol H_2_O m^−2^ s^−1^ in 2018, while the range was 0.58–0.76 mmol H_2_O m^−2^ s^−1^ in 2019. The PP did not affect this parameter in the three irrigation regimes. The gs reduced by 12% in 2018 and 21% in 2019, a key parameter restricting photosynthesis under RF conditions (Massacci et al., 2008; Zhang et al., 2011; Araújo et al., 2019). Similarly, the water losses through E were reduced by 17% in 2018 and 22% in the 2019 seasons, respectively. However, the ETR under RF conditions was higher by 10% and 11% in 2018 and 2019, respectively. Higher ETR is believed to be due to the enhanced operating efficiency of PSII rather than a decrease in the photosynthesis rate in RF crops. This corroborates that ΦPSII increased by 16 and 14% in the 2018 and 2019 seasons, respectively. A similar study conducted in MS reported that leaves of dryland cotton have higher ΦPSII than that of the well-irrigated crop (Pettigrew, 2004). However, other chlorophyll fluorescence parameters, such as NPQ and ΦNPQ, have been recorded significantly higher in RF crops *vis a vis* an irrigated crop. For example, NPQ was higher by 35 and 21% in 2018 and 2019, respectively, while ØNPQ recorded 42 and 50% enhanced levels in 2018 and 2019, respectively. The greater non-photochemical quenching in RF crops is expected to limit energy dissipation, thereby reducing heat damage to the photosynthetic apparatus (Massacci et al., 2008; Gilbert et al., 2011; Slattery et al., 2017). However, the QP reduced in RF crops by 25% in 2018 and 28% in 2019 is probably believed to be a mechanism for reducing electron pressure and increasing open PSII reaction center efficiency by altering redox potential (Massacci et al., 2008; Araújo et al., 2019; Poorter et al., 2022). In contrast, the carbon losses increased by enhanced dark respiration in rainfed crops to the extent of 13% in 2018 and 22% in 2019, thus affecting the cotton lint yields. It was reported earlier by Pinnamaneni et al. (2020a) that the average lint yields in the irrigation and planting geometry combinations were 1,779 kg ha^−1^ in AFI under SR; 2,029 kg ha^−1^ in AFI under TR; 1,803 kg ha^−1^ in SFI under SR; 2,082 in kg ha^−1^ in SFI under TR; 1,573 kg ha^−1^ in RF under SR; and 1,788 kg ha^−1^ in RF under TR.

**Table 3:** Analysis of variance (*F* and *P* values^a^) for the effect of year (Y), planting pattern (PP), and irrigation levels (I) and their interaction on cotton photosynthesis and chlorophyll fluorescence parameters.

**Effect**	**DF**	**LCP**		**LSP**		**AQE**		**A**		**gs**		**E**		**ETR**		**ΦPSII**		**QP**		**NPQ**		**ΦNPQ**		**R**_**d**_	
		***F***	***P***	***F***	***P***	***F***	***P***	***F***	***P***	***F***	***P***	***F***	***P***	***F***	***P***	***F***	***P***	***F***	***P***	***F***	***P***	***F***	***P***	***F***	***P***
Year (Y)	1	13.56	ns	31.45	ns	1.25	^*^	236	^**^	22.82	^**^	15.08	^**^	65.25	^**^	8.46	^**^	5.32	^*^	2.62	^*^	1.68	^*^	2.54	^*^
PP	1	7.65	ns	12.65	ns	0.96	ns	8.69	ns	20.12	ns	5.63	^*^	14.54	ns	6.48	ns	3.42	ns	1.64	ns	1.44	ns	9.82	ns
Y*PP	1	4.21	ns	14.22	ns	1.22	ns	2.82	ns	2.49	ns	2.67	ns	6.36	ns	1.49	ns	1.33	ns	1.09	ns	1.09	ns	1.09	ns
Irrigation (I)	2	22.65	^*^	76.49	^*^	8.87	^*^	168	^**^	105.64	^**^	185	^**^	256	^**^	11.93	^**^	12.21	^**^	9.67	^**^	11.25	^**^	21.44	^*^
Y*I	2	11.57	ns	14.35	^*^	3.54	ns	32.68	ns	11.57	ns	18.36	ns	44.56	^*^	9.32	^*^	1.98	ns	0.67	ns	1.02	ns	0.74	ns
PP^*^I	2	3.42	ns	13.48	ns	1.86	ns	2.46	ns	2.74	^*^	14.66	^**^	12.57	ns	8.19	ns	0.62	ns	0.24	ns	0.31	ns	0.27	ns
PP^*^I^*^Y	2	1.68	ns	5.98	ns	1.55	ns	1.69	ns	1.65	ns	4.52	ns	8.53	ns	0.55	ns	0.18	ns	0.26	ns	0.14	ns	0.13	ns
Residuals		1.24	ns	4.96	ns	0.28	ns	368		1.245		0.324		0.214		0.033		1.223		0.042		0.073		0.013	

a* *Significance at P ≤ 0.05*;

** *Significance at P ≤ 0.001; ns, not significant*.

*LCP, light compensation point (μmol m^−2^ s^−1^), LSP, light saturation point (μmol m^−2^ s^−1^); AQE, apparent quantum efficiency; Amax, Light saturated net photosynthesis (μmol CO_2_ m^−2^ s^−1^); gs, stomatal conductance (mmol H_2_O m^−2^ s^−1^); E, transpiration (mmol H_2_O m^−2^ s^−1^); ETR, electron transport rate (μmol m^−2^ s^−1^); ΦPSII, quantum efficiency of primary PSII photochemistry; QP, photochemical quenching; NPQ, non-photochemical quenching; ΦNPQ, quantum efficiency of non-photochemical quenching; R_d_, dark respiration (μmol m^−2^ s^−1^)*.

*The experiment was conducted in Stoneville, MS, USA, in 2018 and 2019*.

### Comparison Between Soybean and Cotton Photosynthetic Parameters

Although both soybean and cotton are C3 crops, similar trends for different photosynthetic and chlorophyll fluorescence parameters were observed. Still, the extent of differences in regulatory photosynthetic traits and their impact on crop productivity appears to be different. The LCP reduction in soybean was 50% while it was only 20% in cotton under RF conditions. This is probably attributed to paraheliotropic leaf movement in soybean and diaheliotropic movement in cotton under abiotic stress, corroborating with previous reports (Zhang et al., 2011; Hussain et al., 2020). About, 12 and 17% decrease in the photosynthetic rate was observed in RF soybean while the decrease is limited to 9% and 6% in RF cotton during the 2018 and 2019 seasons, respectively. The decrease in photosynthetic rate in soybean is significantly higher than that of cotton under RF compared to irrigated (AFI and SFI) crops. However, a similar decrease in gs and E was observed in the RF condition in both crops. At the same time, the increase in ETR was sharp in RF cotton compared to that of RF soybean (average increase of 10.5% in cotton while 7% in soybean).

Similarly, the NPQ and ΦNPQ, which play a critical role in protecting PSI and PSII reaction centers by dissipating excess energy, recorded an average increase of 39% and 46%, respectively, in RF soybean. In comparison, a 28 and 30% rise was observed in RF cotton for NPQ and ΦNPQ, respectively. These findings are in confirmation with that of earlier reports (Kitao and Lei, 2007; Massacci et al., 2008) but differ from the observations on RF cotton response wherein the quantum yield of PSII was increased, and the regulated non-photochemical energy dissipation was decreased under a water-limited environment (Zhang et al., 2011). This discrepancy could probably be due to the differences in the degree of soil moisture stress and cultivar response. Further, it can be noted that plants dissipate excess solar radiation by triggering NPQ to maintain optimal rates of photosynthesis and provide the plant against oxidative damage (Mishanin et al., 2016). Another study on pot-grown cotton revealed that the PSII quantum yield of photochemistry may or may not be affected by drought in the vegetative stage, subject to drought intensity (Ennahli and Earl, 2005). However, in the case of QP and ΦPSII, there appears to be a similar impact on both RF crops compared to the irrigated crop. The above observations in regulating photosynthetic and chlorophyll fluorescence parameters under The average decrease in soybean grain yield was 16%, while the cotton lint was reduced by 14% (Pinnamaneni et al., 2020a,b).

## Conclusion

It appears that the photosynthetic and chlorophyll fluorescence parameters under diverse irrigation regimes in both soybean and cotton were not affected by alterations in planting geometry, despite the reported higher yields in the TR system. The decrease in LSP, LCP, and Amax in RF crops appears to be relatively lower in cotton than that of soybean, indicating either lower water requirement or better tolerance to moisture deficit. However, soybean recorded decreased AQE while cotton exhibited higher AQE under RF conditions. The results of this study indicated preferential use of non-photochemical energy dissipation in soybean while cotton uses both photochemical and non-photochemical energy dissipation to protect PSI and PSII centers and ETR, although they fall under the C3 species. Detailed studies with diverse moisture stress intensities coupled with physiological and anatomical parameters during multiple crop growth stages will help further delineate the mechanistic role of photosynthetic and chlorophyll fluorescence pathways in determining soybean and cotton productivities.

## Data Availability Statement

The raw data supporting the conclusions of this article will be made available by the authors, without undue reservation.

## Author Contributions

SP: conceptualization, experiment design, data collection, project administration, analysis, writing the manuscript, and visualization. SA: conceptualization, project administration, manuscript review and editing, resources, and visualization. KR: conceptualization, project administration, manuscript review, editing, and resources. All authors contributed to the article and approved the submitted version.

## Funding

This work was supported by the U. S. Department of Agriculture, Agriculture Research Service through project number 6066-22000-089-000D.

## Author Disclaimer

Trade names are necessary to report factually on available data; however, the USDA neither guarantees nor warrants the standard of the product or service. The use of the name by USDA implies no approval of the product or service to exclude others that may also be suitable.

## Conflict of Interest

The authors declare that the research was conducted in the absence of any commercial or financial relationships that could be construed as a potential conflict of interest.

## Publisher's Note

All claims expressed in this article are solely those of the authors and do not necessarily represent those of their affiliated organizations, or those of the publisher, the editors and the reviewers. Any product that may be evaluated in this article, or claim that may be made by its manufacturer, is not guaranteed or endorsed by the publisher.
